# Barriers and facilitators to the implementation and scale up of differentiated service delivery models for HIV treatment in Africa: a scoping review

**DOI:** 10.1186/s12913-022-08825-2

**Published:** 2022-11-28

**Authors:** Yihalem Abebe Belay, Mezgebu Yitayal, Asmamaw Atnafu, Fitalew Agimass Taye

**Affiliations:** 1 Department of Public Health, College of Health Sciences, Debre Markos University, Debre Markos, Ethiopia; 2 Department of Health Systems and Policy, Institute of Public Health, College of Medicine and Health Sciences, University of Gondar, Gondar, Ethiopia; 3 Department of Accounting, Finance, and Economics, Griffith University, Brisbane, Australia

**Keywords:** Differentiated service delivery, Implementation, Scale up, ART, Africa, Review

## Abstract

**Background:**

In the face of health-system constraints, local policymakers and decision-makers face difficult choices about how to implement, expand and institutionalize antiretroviral therapy (ART) services. This scoping review aimed to describe the barriers and facilitators to the implementation and scale up of differentiated service delivery (DSD) models for HIV treatment in Africa.

**Methods:**

PubMed, Web of Science, Embase, Scopus, CINAHL, Global Health, Google, and Google Scholar databases were searched. There was no start date thereby all references up until May 12, 2021, were included in this review. We included studies reported in the English language focusing on stable adult people living with human immune deficiency virus (HIV) on ART and the healthcare providers in Africa. Studies related to children, adolescents, pregnant and lactating women, and key populations (people who inject drugs, men having sex with men, transgender persons, sex workers, and prisoners), and studies about effectiveness, cost, cost-effectiveness, and pre or post-exposure prophylaxis were excluded. A descriptive analysis was done.

**Results:**

Fifty-seven articles fulfilled our eligibility criteria. Several factors influencing DSD implementation and scale-up emerged. There is variability in the reported factors across DSD models and studies, with the same element serving as a facilitator in one context but a barrier in another. Perceived reduction in costs of visit for patients, reduction in staff workload and overburdening of health facilities, and improved or maintained patients’ adherence and retention were reported facilitators for implementing DSD models. Patients’ fear of stigma and discrimination, patients’ and providers’ low literacy levels on the DSD model, ARV drug stock-outs, and supply chain inconsistencies were major barriers affecting DSD model implementation. Stigma, lack of model adoption from providers, and a lack of resources were reported as a bottleneck for the DSD model scale up. Leadership and governance were reported as both a facilitator and a barrier to scaling up the DSD model.

**Conclusions:**

This review has important implications for policy, practice, and research as it increases understanding of the factors that influence DSD model implementation and scale up. Large-scale studies based on implementation and scale up theories, models, and frameworks focusing on each DSD model in each healthcare setting are needed.

**Supplementary Information:**

The online version contains supplementary material available at 10.1186/s12913-022-08825-2.

## Background

Africa bears the highest global human immunodeficiency virus (HIV)/acquired immunodeficiency syndrome (AIDS) burden, with over two-thirds of all HIV-positive people (25.7 million) residing in this developing region with severe gaps in access to HIV services (prevention, diagnosis, treatment, and care) [1]. The Joint United Nations Programme on HIV/AIDS (UNAIDS) set 90–90–90 goals for 2020 in response to the HIV epidemic, aiming to ensure that 90% of all individuals living with HIV know their HIV status, 90% of all persons with confirmed HIV infection receive sustained ART, and 90% of all people getting ART have viral suppression. A new 95-95-95 target has been set for 2030 [2]. To achieve the 90-90-90 goals, the World Health Organization (WHO) released ART guidelines recommending a “treat-all” approach, whereby all HIV-positive populations and age groups are eligible for ART [3].

In 2015, the WHO recommended differentiated models of care, emphasizing the need to strengthen the continuum of HIV care and improve service quality and access, adherence and retention, clinical outcomes, efficiency, and cost of services, particularly in high-prevalence countries [3, 4]. The differentiated HIV treatment for clinically stable patients is a component of DSD models for HIV which focus on the second and third 90-90-90 targets [5].

Differentiated HIV treatment models aim to put people at the center of antiretroviral delivery and are characterized by four components: i) types of services delivered; (ii) location of service delivery; (iii) provider of health services; and (iv) frequency of health services [4, 5].

The DSD models for HIV treatment can be described within four categories. In healthcare worker-managed groups, clients receive their ART refills in a group and either a professional or a lay healthcare staff member manages this group. The groups meet within and/or outside of healthcare facilities. In client-managed group models, clients receive their ART refills in a group in which clients meet outside of health care facilities and manage and run the refills themselves. In facility-based individual models, ART refill visits are separated from clinical consultations. When clients have an ART refill visit, they bypass any clinical staff or adherence support and proceed directly to receive their medication. For out-of-facility individual models, ART refills and, in some cases, clinical consultations are provided to individuals outside of healthcare facilities, for example, community pharmacies, outreach models, and home delivery [6].

To achieve the promise of DSD, model adoption, implementation, scale-up, and evaluation are necessary processes [7]. Since 2016, numerous countries, particularly in sub-Saharan Africa and for adults established on ART, have embraced and scaled up DSD as part of national policy [8]. The optimal mix of DSD models for HIV treatment at the national level is specific to each country’s context [9]. The effective implementation and scale-up of DSD models is an ongoing challenge in Africa. The term implementation in relation to health interventions is defined as “the use of strategies to adopt and integrate evidence based health interventions and change practice patterns within specific settings” [10]. The WHO/ ExpandNet defines scale up as: “deliberate efforts to increase the impact of successfully tested health innovations to benefit more people and to foster policy and program development on a lasting basis” [11].

Understanding factors that influence the implementation and scale up of DSD models is a considerable research and practice benefit to get the picture of why DSD model implementation and scale up can succeed or fail. Several studies assessing the barriers and facilitators for DSD implementation and scale up have been conducted in Africa although a little attempt has been made previously to map the available research findings using a scoping review format. Previous literature reviews lacked particular focus and in-depth investigation of the factors influencing the DSD interventions implementation and scale up [12–16]. Therefore, this study aimed to review the available research reporting on barriers and facilitators for the effective implementation and scale-up of DSD models in Africa, to guide policymakers, program managers, and practitioners as they implement, expand and institutionalize ART services.

## Methods

This scoping review follows the Joanna Briggs Institute (JBI) methodology for scoping review [17]. We didn’t register the protocol for this study since scoping reviews are currently ineligible for registration in the PROSPERO database. However, we strictly followed the PRISMA ScR checklist [18] to check our scoping review conforms to this reporting standard.

### Eligibility criteria

#### Population

This review is comprised of evidence involving stable adult people living with HIV taking antiretrovirals (ARVs), and the healthcare workers providing ART services. Stable adult HIV-positive clients with a controlled chronic disease were also included. However, the evidence related to children, adolescents, pregnant and lactating women, and key populations (people who inject drugs, men having sex with men, transgender persons, sex workers, and prisoners) were excluded due to special criteria for defining clinically stable clients, and key considerations for social and legal issues in accessing ART services.

#### Concept

Studies that reported the barriers and facilitators to the implementation and scale-up of DSD models were included.

#### Context

This review included only studies conducted in Africa, where there is a high burden of HIV and limited public health resources, with a varied range of communities and cultures.

#### Types of the sources of evidence

The source of information is comprised of studies published in peer-reviewed journals (primary research studies, systematic reviews, and non-systematic reviews), conference proceedings, and unpublished theses and dissertations. Only the English language-based studies were included because of limited resources for the translation of studies conducted in languages other than English. There was no start date thereby all studies up until May 12, 2021, were included in this review. In addition, studies reporting effectiveness, cost, cost-effectiveness, and pre or post-exposure prophylaxis were excluded since these types of studies didn’t directly evaluate the barriers and facilitators affecting the implementation and scale up of specific DSD models.

### Search strategy

A three-phase search strategy was carried out using databases including PubMed, Web of Science Core Collection, Embase, Scopus, CINAHL, and Global Health. The first phase was an initial limited search of PubMed to identify relevant records.

Secondly, the search strategy was developed according to the previous phase using all identified keywords and index terms, and it was customized for each included information source. A comprehensive search strategy and set of search terms is contained in Additional file 1. Search terms included

1. patient* OR client* OR provider*
2. “human immunodeficiency virus” OR “human immunodeficiency virus infection” OR HIV OR “antiretroviral treatment” OR “antiretroviral therapy” OR “antiretroviral therapy, highly active” OR “highly active antiretroviral therapy” OR HAART OR ART
3. “patient-centred care” OR “patient-centered care” OR “community supported models” OR “adherence club*” OR “task shifting” OR “community ART distribution” OR “community ART delivery” OR “community ART refill” OR “community client lead ART-delivery” OR “facility fast track” OR “quick pick-up” OR “differentiated care” OR “differentiated service” OR “differentiated intervention” OR “decentrali?ed. care” OR “decentrali?ed. service” OR “decentrali?ed. intervention” OR “community care” OR “community service” OR “community intervention” OR “differentiated model*” OR down-referr* OR out-of-clinic
4. experience* OR attitude* OR perception* OR learning OR Barrie* OR challeng* OR facilitator* OR enabler* OR benefit* OR success* OR constrain* OR difficult* OR enhanc* OR influen* OR interfer* OR motivat* OR obstruct* OR problem* OR promot* OR restrain* OR restrict* OR implement* OR uptake OR adopt* OR adapt* OR accept* OR react* OR appropr* OR feasib* OR fidelity OR sustain* OR modification OR scale-up OR scaling-up OR scale up OR scale-out OR expan* OR replica* OR exten* OR institutionali?ation OR maintain OR continue*
5. Combining all 54 countries in Africa by the Boolean operator ‘OR’

Finally, the reference lists of all the included studies were screened for additional records. Grey literature was also searched from relevant HIV related conference databases (International AIDS Society (IAS), Conference on Retroviruses and Opportunistic Infections (CROI), South African AIDS Conference (SAAIDS), Southern African HIV Clinicians Society (SAHIVSOC), European AIDS Conference (EACS), INTEREST Conference, Zambia Health Research Conference (ZHRC), Asia Pacific AIDS & Co-infections Conference (APACC), and International Conference on AIDS and STI’s in Africa (ICASA)) via Google and Google scholar search engines.

### Study selection

All retrieved studies were exported to Endnote version 9 (Thomson Reuters, London) reference manager, and duplications were carefully removed. Two investigators (YAB and FAT) independently screened the titles and abstracts of studies identified from each database using the inclusion and exclusion criteria. Any disagreements that arose between the reviewers were resolved through discussion and the involvement of the third reviewer (MY). Then, full texts were retrieved for all studies that passed the title and abstract screening.

**Fig. 1:**
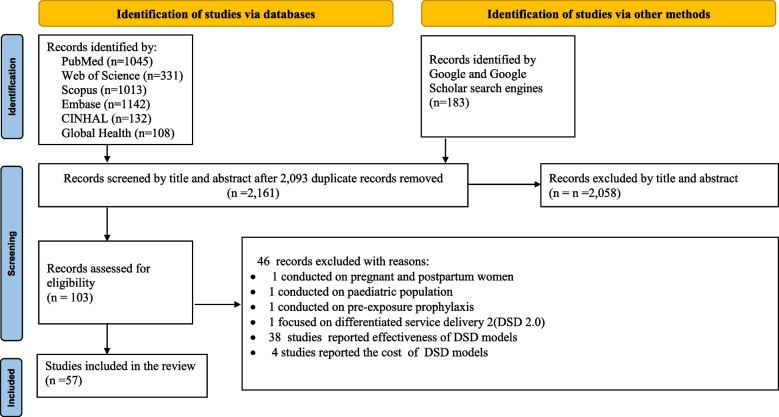
PRISMA flow diagram of the included studies

Our search of databases and other sources yielded 4254 records. After removing 2093 duplicate records, 2161 records were screened at the title and abstract level, resulting in 103 records being evaluated for eligibility. From these, 46 records were excluded (38 reported effectiveness of DSD models, 4 reported cost of DSD models, 1 conducted with pregnant and postpartum women, 1 conducted with the pediatric population, 1 conducted with pre-exposure prophylaxis, and 1 focused with DSD 2.0). Ultimately, 57 articles were included in the scoping review (Fig. 1).

### Data extraction

The data from full texts of included studies were extracted using a JBI data extraction template in the form of customized Microsoft Excel [17]. Two independent reviewers (YAB and MY) extracted the data and cross-checked it to ensure consistency. Any discrepancies that arose between the reviewers were solved by a discussion with a third reviewer (AA). The reviewer (YAB) contacted the corresponding author(s) for further information whenever pertinent data was missed from the included studies. According to the JBI Reviewers manual [19], descriptive data on the author(s), year, types of evidence source, publication type, country of origin, aims, study design, study population, concept, context, and key findings, in line with the review questions, were extracted (Additional file 2).

### Analysis and presentation of results

A descriptive analysis was done in this scoping review. Barriers and facilitators reported in included studies were summarised. The identified barriers and facilitators in this study were clustered according to implementation and scale up aspects of different DSD interventions.

Implementation related barriers and facilitators were further clustered according to the four categories of DSD models for HIV treatment described in practice and the literature: group models managed by healthcare workers; group models managed by clients; individual models based at facilities; and individual models based out of facilities [5]. We have analyzed factors identified from the perspectives of patients and providers and presented these separately in each DSD model category. The scale up related barriers and facilitators were further categorized based on the components of the Health System Dynamics Framework [20].

The search results were presented in a Preferred Reporting Items for Systematic Reviews and Meta-Analyses(PRISMA) Flow Diagram for the scoping review process [21], tables listing the results, and a descriptive summary using texts per the review questions.

## Results

### Characteristics of included studies

Of all included studies, more than one-third (21) of studies were conducted in South Africa only [12, 22–41], and five of them were undertaken in sub-Saharan Africa [13–16, 42]. Nearly two-thirds (33) of the included studies were primary published articles [22, 23, 25, 27, 30, 32–39, 43–60] followed by nearly one-fifth (12) of conference abstracts [29, 31, 41, 55, 61–69]. The studies included in the scoping review were multi-methods comprising mixed method, qualitative and quantitative types of studies. Nineteen (33.3%) of the studies were descriptive qualitative [22, 24, 28, 29, 32, 33, 39, 47, 49, 52–56, 58–60, 62, 68] (Table 1).

**Table 1:** Summary characteristics of studies included in the scoping review

Author and year	Country	Types of evidence source	Aims	Study design	Information source
Francis et al., 2017 [61]	Tanzania	Conference abstract/ Unpublished	To ascertain the acceptability and logistical feasibility of community health worker-led ART home-delivery in Dar es Salaam	Mixed method	Patients and healthcare providers
Mashungu et al., 2018 [62]	Zimbabwe	Conference abstract/ Unpublished	To explore patient and service provider’s acceptability of community ART refill groups in Zimbabwe	Descriptive qualitative	ART service providers and stable patients enrolled in community ART refill groups
Tshuma et al.,2017 [22]	South Africa	Primary study/Published	To assess acceptability, enablers, and barriers to rolling out community-based adherence clubs in South Africa	Descriptive qualitative	Nurses, club managers, data capturers, pharmacists, and pharmacy assistants who had been involved in facility-based treatment adherence clubs
Rasschaert et al.,2014 [43]	Mozambique	Primary study/Published	To analyze the evolution of the community ART group model from 2008 to 2012	Mixed method	Patients on ART in groups and in individual care, Nurses, Medecins Sans Frontieres Counsellors, Health authorities, and Medecins Sans Frontieres implementers
Asieba et al., 2021 [44]	Nigeria	Primary study/Published	To assess the feasibility, acceptability, and outcomes of the community pharmacy-based ART refill model in Nigeria	Retrospective analysis	ART clients and Community Pharmacists
Flamig et al., 2019 [12]	South Africa	Scoping literature review/Published	To identify factors that enable or jeopardize the sustainability of the Adherence Club model in the Western Cape of South Africa	Scoping literature review	Articles on ART adherence clubs in South Africa
Mukumbang et al., 2019 [23]	South Africa	Primary study/Published	To test a theory on how and why the adherence club intervention works and in what health system context(s) in a primary healthcare facility in the Western Cape Province, South Africa	Realist evaluation	Nurses, counselors (club facilitators), and patients (both current and former club members)
Laga et al., 2017 [42]	sub-Saharan Africa	Dissertation project/Unpublished	To describe the challenges with decentralization to community-based primary health care facilities, in Tete, Mozambique	Mixed method	Community ART groups
Bemelmans et al., 2014 [45]	Malawi, South Africa, DRC, and Mozambique	Primary study/Published	To describe a number of community-supported models of ART delivery developed by Medecins Sans Frontieres together with Ministries of Health in public health facilities in sub-Saharan Africa	Routine program data analysis	Appointment spacing for clinical and drug refill visits in Malawi, peer educator-led ART refill groups in South Africa, community ART distribution points in the Democratic Republic of Congo, and patient-led community ART groups in Mozambique
Ndlovu et al., 2020 [24]	South Africa	Master’s thesis project/Unpublished	To explore and compare the experiences of patients in three differentiated care models (Facility Adherence Clubs, Community Adherence Club, and Quick Pharmacy Pick-up) in a community health care facility in a township in Cape Town, South Africa	Descriptive qualitative	People living with HIV (18 years plus) receiving ART in a differentiated ART delivery model
Ssonko et al., 2017 [46]	South Sudan,the Central African Republic, and the Democratic Republic of Congo	Primary study/Published	To review the implementation of differentiated HIV care and treatment approaches in Medecins Sans Frontieres-supported programs	Descriptive analysis	Routinely Program data
Christ et al., 2020 [70]	Zimbabwe	Manuscript (preprint)/Unpublished	To assess the availability of differentiated ART models and the experience of health care professionals and clients in the rural district of Bikita, Masvingo Province, Zimbabwe	Mixed-method	ART clients and healthcare providers
Sharer et al., 2019 [25]	South Africa	Primary study/Published	To (a) gain an in-depth understanding of perceived implementation barriers and enablers for differentiated ART delivery models in South Africa and (b) explore pragmatic concerns from program implementers and nurses related to sustainability and integration into existing ART programs to support treatment scale-up	Formative evaluation	Program implementers, nurses, and other health care providers
Duffy et al., 2019 [47]	South Africa, Uganda, and Zimbabwe	Primary study/Published	To describe differentiated treatment distribution models and identify enablers, barriers, and benefits of the models by synthesizing findings from multistakeholder interviews and focus group discussions with participants in South Africa, Uganda, and Zimbabwe	Descriptive qualitative	High-level policymakers/influencers, Program designers, managers, and implementers, Health service providers, and Patients
Hagey et al., 2018 [13]	sub-Saharan Africa	Scoping review/Published	To describe the range of HIV care for stable patients within the differentiated care framework	Scoping literature review	Manuscripts on differentiated HIV care
Kuchukhidze et al., 2019 [14]	sub-Saharan Africa	A review of the grey literature/Unpublished	To conduct a comprehensive search of unpublished reports and other data sources posted online or directly from DSD implementers	Review of grey literature	Grey documents
Long et al., 2020 [15]	sub-Saharan Africa	A rapid systematic review/Unpublished	To conduct a rapid review of the most recent peer-reviewed reports of the outcomes of DSD model implementation in sub-Saharan Africa	Rapid systematic review	Peer-reviewed reports of the outcomes of DSD model implementation
Huber et al., 2020 [48]	Malawi, South Africa, and Zambia	Primary study/Published	To describe the diversity of DSD models being implemented	Cross-sectional	DSD model implementing organizations
Mulenga et al., 2019 [63]	Zambia	Conference abstract/ Unpublished	To describe the model and early results herein	Descriptive analysis	People living with HIV on ART
Adjetey et al., 2019 [49]	Ghana	Primary study/Published	To explore the possible predictors and acceptability of Community-based health service provision among people living with HIV accessing ART services at the Cape Coast Teaching Hospital in Ghana	Descriptive qualitative	People living with HIV accessing ART services
Liu et al., 2021 [26]	South Africa	Manuscript (preprint)	To describe the expansion of central chronic medicines dispensing and distribution to a national scale	Mixed methods evaluation	Patients with chronic disease, including HIV
Wilkinson et al., 2016 [27]	South Africa	Primary study/Published	To describe the implementation of the Adherence Club model across the Cape Metro health district in Cape Town, South Africa, between January 2011 and March 2015	Data aggregate of the monthly monitoring report	Facilities offering adherence clubs and patients receiving ART care in the adherence club model
Dudhia and Kagee, 2015 [28]	South Africa	Primary study/Published	To document the experiences of patients attending adherence clubs and health care workers at clinics where clubs were operating in South Africa	Descriptive qualitative	ART adherence club members and healthcare workers
Kizito and Sabiti, 2021 [50]	Uganda	Primary study/Published	To describe the factors associated with the uptake of community client-led ART delivery model at Mulago Adult HIV clinic in Mulago National Referral Hospital	Mixed-method	Adult HIV patients who are stable on ART and receiving ART from Mulago adult HIV clinic and service providers
Venables et al., 2017 [29]	South Africa	Conference abstract/Unpublished	To explore perceptions of clubs amongst members and non-members in two sites in Cape Town, South Africa	Descriptive qualitative	People living with HIV(current club members, eligible patients who had never joined a club, and club members who had been returned to routine care)
Roy et al., 2018 [64]	Zambia	Conference abstract/Unpublished	To evaluate the implementation and effectiveness of urban adherence clubs in Zambia using a randomized study design	Randomized controlled trial	HIV-positive patients and healthcare workers
Pasipamire et al., 2016 [65]	Swaziland	Conference abstract/Unpublished	To assess the feasibility of implementing community ART models in Swaziland	Cohort	Stable patients on ART
Grimsrud et al., 2015 [30]	South Africa	Primary study/Published	To describe the implementation of community-based adherence clubs at a large, public-sector facility in peri-urban Cape Town, South Africa	Descriptive analysis	Community-based adherence clubs
Wilkinson et al., 2015 [31]	South Africa	Conference abstract/Unpublished	To describe scale up of adherence clubs between Jan 2011-March 2015 in the Cape Metro, South Africa	Descriptive analysis	Stable ART patients
Zakumumpa et al., 2021 [71]	Uganda	Manuscript (preprint)/Unpublished	To assess the extent of uptake of differentiated ART models and to describe barriers to uptake of either facility-based or community-based models in a national sample of health facilities in Uganda	Mixed-method	Health facilities, and ART clinic managers
Zakumumpa et al., 2017 [51]	Uganda	Primary study/Published	To identify modifications to ART service delivery models by health facilities in Uganda to sustain ART interventions over 10 years (2004–2014)	Mixed method	Health facilities, and ART clinic managers
Prust, 2017 [72]	Malawi	Process evaluation report/Unpublished	To explore the process and guidelines for implementation, the extent of implementation in participating facilities, provider and patient perspectives on the models, and costs of the differentiated service delivery models in Malawi	Process evaluation	Health facilities, healthcare workers, and patients
Prust et al., 2017 [66]	Malawi	Conference abstract/Unpublished	To assess the extent to which patients are accurately differentiated as eligible or ineligible for multimonth scripting and explore potential causes of inaccurate patient differentiation in Malawi	Mixed -method	Health facilities, health workers, and clinic management
Attah et al., 2018 [67]	Nigeria	Conference abstract/Unpublished	To assess the impact of Nigeria’s antiretroviral multimonth scripting on public health service delivery, infrastructure, and supply chain management systems across “high volume” ART clinics	Descriptive analysis	Clinic attendance records and ART refill providers
Keene et al., 2020 [32]	South Africa	Primary study/Published	To explore patient, healthcare worker, and key informant experiences and perceptions of extending ART refills to 6 months in adherence clubs in Khayelitsha, South Africa	Descriptive qualitative	Patients, healthcare workers, and Key informants
Prust et al., 2018 [52]	Malawi	Primary study/Published	To understand the challenges and successes of implementing these models of care and the process of patient differentiation in Malawi	Descriptive qualitative	Patients and health workers
Venables et al., 2019 [33]	South Africa	Primary study/Published	To explore patient experiences of clubs in two sites in Cape Town, South Africa	Descriptive qualitative	Patients
De Jager et al., 2018 [34]	South Africa	Primary study/Published	To investigate treatment adherence and patient satisfaction of stable HIV patients on ART in ART adherence clubs and clinics in South Africa	Cross-sectional	Stable HIV patients on ART in ART adherence clubs and clinics
Mukumbang et al., 2018 [35]	South Africa	Primary study/Published	To determine how, why, for whom, and under what health system context the adherence club intervention works (or not) in real-life implementation in South Africa	Realist evaluation	Doctors, Adherence club nurses, Adherence club counselors/club facilitators, Patients in clubs, and Former club patients
Mudavanhu et al., 2020 [36]	South Africa	Primary study/Published	To explore patient acceptability and attitudes towards community and clinic-based adherence clubs in South Africa	Mixed method	Patients on ART
Phiri et al., 2021 [53]	Zambia	Primary study/Published	To understand providers’ perceptions of the benefits and challenges of six-month versus three-month ART dispensing in Zambia	Descriptive qualitative	Multimonth dispensing providers
Rasschaert et al., 2014 [54]	Mozambique	Primary study/Published	To evaluate the relevance, dynamic, and impact of the community ART group model on patients, their communities, and the healthcare system in Tete, Mozambique	Descriptive qualitative	Patients on ART, nurses, Medecins Sans Frontieres lay counselors, and health authorities
Hubbard et al., 2020 [55]	Malawi	Primary study/Published	To explore client and provider experiences with multimonth dispensing in Malawi as part of a cluster-randomized trial evaluating 3- versus 6-month ART dispensing	Descriptive qualitative	Providers and clients
Bock et al., 2019 [37]	South Africa	Primary study/Published	To report on clinical outcomes among ART clients attending adherence clubs and client experiences and healthcare worker perceptions of factors key to successful adherence club implementation in the Cape Winelands District, South Africa	Crossectional and retrospective cohort	Clients and healthcare workers
Roy et al., 2019 [16]	sub-Saharan Africa	Review/Published	To review the available published evidence on the implementation of DSD and suggest further health systems innovations needed to maximize the public health impact of DSD and future implementation science research directions in this expanding field	Review of literature	Published evidence
Bochner et al., 2019 [56]	Zimbabwe	Primary study/Published	To assess the perceived effects of this new national service delivery model in Zimbabwe	Descriptive qualitative	Healthcare workers and ART clients
MacGregor et al., 2018 [38]	South Africa	Primary study/Published	To explore the challenges associated with taking to scale a pilot that began as a relatively simple innovation by a non-governmental organization	Mixed -method	Health facilities, patients receiving ART in an adherence club, and staff working with ART adherence clubs
Decroo et al., 2013 [57]	Mozambique	Primary study/Published	To describe the stepwise implementation and roll-out of Community ART groups in Mozambique	Descriptive study	Community ART groups
Rasschaert et al., 2014 [58]	Mozambique	Primary study/Published	To identify factors influencing the sustainability of the community ART group model in Mozambique	Descriptive qualitative	Patients on ART, nurses, Medecins Sans Frontieres lay counselors, health authorities, and Medecins Sans Frontieres community ART group implementers
Dorward et al., 2020 [39]	South Africa	Primary study/Published	To explore how centralized chronic medication dispensing and distribution influences engagement in HIV care	Descriptive qualitative	Clients receiving ART and healthcare workers
Zakumumpa et al., 2020 [59]	Uganda	Primary study/Published	To explore patients’ and HIV service managers’ perspectives on barriers to the implementation of Differentiated ART service delivery	Descriptive qualitative	National-level HIV program managers, district health team leaders, representatives of United States President’s Emergency Plan for AIDS Relief implementing organizations, and ART clinic in-charges
Hubbard et al., unknown year of presentation [68]	Malawi	Conference abstract/Unpublished	To explore client and provider experiences with the implementation of multi-month dispensing in Malawi as part of the INTERVAL study	Descriptive qualitative	ART client and provider
Mukumbang et al., 2018 [35]	South Africa	Primary study/Published	To develop a refined program theory explicating how, why, for whom, and under what health system contexts the adherence club intervention works (or not)	Realist evaluation	Program designers and managers, available studies on group-based ART adherence support models in sub-Saharan Africa, and social, cognitive, and behavioral theories that have been applied to explain adherence to ART
Roy et al., 2017 [69]	Zambia	Conference abstract/Unpublished	1. To describe the uptake of the community adherence group model in Zambia using an implementation cascade for individuals offered community adherence groups 2. To identify adaptations to the community adherence group model during early implementation in Zambia	Program evaluation	Stable patients engaged in the community adherence group model
Pellecchia et al., 2017 [60]	Malawi	Primary study/Published	To assess the benefits and challenges of community adherence groups from patients’ and healthcare workers’ perspectives	Descriptive qualitative	Community adherence group members, ART patients eligible for community ART refill groups who remained in conventional care, former community ART refill group members who returned to conventional care, and healthcare workers responsible for providing HIV care
Mukumbang et al., 2019 [40]	South Africa	Document review/Published	To review the effectiveness of the rollout of the antiretroviral adherence club intervention in South Africa to date through an implementation research framework	Document review	Documents of the adherence club program
Davey et al., 2018 [41]	South Africa	Conference abstract/Unpublished	To evaluate demographic and clinical characteristics and treatment outcomes in patients on differentiated care versus standard care	Crossectional	Documents of the adherence club program

*ART* antiretroviral therapy, *DSD* differentiated service delivery, *HIV* human immune deficiency virus

### Barriers and facilitators to the implementation of DSD models

Forty-two studies have reported findings on the implementation-related barriers and facilitators. The majority of the included studies highlighted both barriers and facilitators in the same study. Some studies however focused solely on the barriers. Across many DSD models, there were common facilitators and barriers. In addition, there is variability in the reported factors across DSD models and studies, with the same element serving as a facilitator in one context but a barrier in another. The following section summarized the influencing factors according to the four categories of DSD models for HIV treatment (Table 2).

**Table 2:** Barriers and facilitators to the implementation of differentiated antiretroviral therapy service delivery in Africa, 2021

Model category	Facility-based individual models	Out of facility-based individual models	Client led groups	Healthcare worker-led groups
Barriers to implementation	❖ Health facilities implement multimoth scripting refill length inconsistently [15, 52] ❖ Fast track refill lack patient-centeredness [24] ❖ Providers concerned with the perceived inability to provide adequate care could feel disconnected from their patients and could miss “silent issues”, doubted patient abilities to adhere to medication [47] ❖ Multimoth scripting could cause patients to be more likely to miss appointments because of a long length of time between schedules [52] ❖ Patients were not coming back to the clinic promptly to report any problems [14, 52, 55] ❖ At multimoth scripting initiation, the number of ARV issues to patients increased; these lead to short term supply risk that required a temporary slowdown of its implementation [67] ❖ Providers lack of information on model implementation [15] ❖ Antiretroviral drug stock-outs and supply chain inconsistencies [14, 15, 52, 59] ❖ Providers were concerned with an increased possibility of medications being misused by patients [52], antiretroviral sharing with family or friends making pill count difficult [14, 55] ❖ Feasibility at the clients level regarding large volume of ART drug storage at home [55] ❖ Patients were concerned with the fear of inadvertent disclosure due to having to store large quantities of medication at home and concerns regarding the safety and storage of medication for prolonged periods at home [14, 47]	▪ Fear of detachment from the formal health system [59] ▪ Fears that prolonged periods without being seen by health workers would imply an inability to access comprehensive care including in the event of opportunistic infections such as Tuberculosis [59] ▪ Patients lack clarity with regard to how models work [14] ▪ Some patients reported a missing dose because their medication was not delivered at home on time [15, 61] ▪ Fear of accidental disclosure and its associated stigma and discrimination [49] ▪ Need for vehicles and fuel to transport health workers into communities [59] ▪ Need for health worker monetary allowances during community visits [59] ▪ The difficulty in finding suitable physical infrastructure in rural settings to designate as outreach points for ART refills [59] ▪ The additional burden due to data collection responsibilities [14] ▪ Frequent drug stock-outs and supply chain problems [14] ▪ Expensive to implement and yet facilities had not received adequate funding and resource facilitation from donors and the government [71]	➢ Patients prefer meeting with the healthcare provider one-to-one to protect confidentiality [14] ➢ Fear of stigma, discrimination, and losing respect as reasons for not joining groups [50, 59, 62] ➢ Some clients expressed a lack of cooperation among individuals as the likely reason why some patients fear forming the community client lead antiretroviral distribution groups since they do not know each other at the beginning and they fear clashing in the community [50] ➢ Some clients reported fear of bad doing through someone else handling their medication as one of the reasons for not joining client lead groups [62] ➢ Fear of detachment from the formal health system [59] ➢ Some patients were dissatisfied with the efficiency of drug pickups [14] ➢ Group leaders of patient groups expressed difficulty in sustaining transport costs to facilities to pick drugs on behalf of their colleagues, and have concerns about identifying ART refill packages for each of their members [59] ➢ Lack of sufficient resources to perform what is expected from them for DSD [14, 59] ➢ Disorganization of medical records [14] ➢ The additional workload involved in packaging and labeling antiretrovirals for each member while decentralization of drug delivery to communities [59, 70] ➢ Difficulty in finding competent and literate leaders of community client lead ART distribution groups [59] ➢ Patients may not seek needed care [15, 56] ➢ Frequent changes in physical addresses among urban clients impeded the running of patient groups of rotating ART refill pick-ups [59] ➢ Low patient literacy of DSD models [59]	• Inadvertent status disclosure [15, 22, 70] • Infrequent clinician visits and needing to find members to join their group [15] • Challenges to ART supply to the adherence clubs [14] • Patients lack clarity with regard to how models work [15] • Inadequate medical recordkeeping [14] • An increase in the probability of many patients defaulting from picking up their medication if adherence clubs are implemented in community venues [22] • Increased burden on staff [14, 15] • Incorrect patient differentiation [14] • Security of medication [15, 22] • ART storage conditions [15, 22] • Infrastructure (space) concerns [15, 22] • Providers concerned with the transportation of the prepacked medication to the distribution sites [15, 22] • Staff shortage [15] • Lack of compensation for staff working off-hours [15] • Lack of staff clarity on eligibility criteria [15] • Lack of staff clarity on the rationale for referral back to the standard of care [15]
Facilitators to implementation	❖ Having comprehensive health checks before taking necessary medications [49] ❖ Perceived higher need for privacy and confidentiality by clients especially for urban and high-income categories [59, 71] ❖ Reduced travel cost [14, 52, 53, 55, 68] ❖ Reduced waiting time [14, 15, 52, 67, 70] ❖ Flexible characteristics of the FTR model(patients could also collect antiretroviral drugs outside of working hours including evening time) [24] ❖ Alleviate issues with absenteeism from work for clinic appointments [52] ❖ Increased time for income-generating activities [55, 68] ❖ Improved freedom for employment and family travel [14] ❖ Improved or maintained adherence [15, 67] ❖ Improved overall patient satisfaction with clinic services [15, 67] ❖ Encourage patients not in care to seek services [52] ❖ A greater sense of personal freedom and normalcy [55, 68] ❖ Having no reports of antiretroviral trade or misuse and unwanted HIV disclosure, and antiretrovirals are easily and safely stored at home [14] ❖ Reduction in staff workload [15, 47, 52, 53, 55, 67, 68] ❖ Reduction in the overburdening of health facilities [15, 47, 52, 53, 67] ❖ Requiring least resource inputs (fast track refill is most practical to implement) [71] ❖ Having no reports of antiretrovirals shortages or expiration [14]	▪ Reduced patient travel cost [14, 49] ▪ Reduction in the overburdening of health facilities [14] ▪ Better care for sicker patients [14] ▪ Role in continuation of care at community pharmacy [44] ▪ Support care retention for established, stable patients on ART [63] ▪ The convenience of accessing medications in the comfort of their own home [49] ▪ Overcame material barriers to attending clinics, changed the meanings associated with collecting ART, and was less disruptive to other social practices in clients’ lives [39]	➢ Increasing group and social support [15, 52, 56] ➢ Reduction in the overburdening of health facilities and higher quality of care for unstable patients [15, 56, 62] ➢ More time spent on patient data compilation and viral load testing to improve monitoring [15, 62] ➢ Reduced transport costs [15, 56, 59, 62, 70] ➢ Have an important role in adherence and defaulter tracing for improved retention [15, 56] ➢ New client lead group members anticipate the benefit of a reduction in facility visits thereby allowing increased focus on productive activities, and group support through livelihood projects, adherence, and defaulter tracing thereby improving retention, lifestyles, and psychological well-being [62]	• Forming community-based patient support structures in the form of support groups and open the door for patient empowerment and self-management [14, 22, 42] • Reduced transport costs [14, 70] • Better linkage to care [14, 22] • Improvement in adherence to treatment [14, 22] • Reduction in defaulter rate and tracking of lost to follow up [14, 22] • Facility decongestion [14, 22] • Reduction of provider burden [14, 22] • Give more opportunities for task-sharing between clinic staff [14, 22] • Promising health outcomes, especially convenient for patients who work [15] • Flexibility to pick up ARTs after the appointment date [15] • Models that allow for family members to pick up antiretrovirals on behalf of the patients are especially convenient [14] • Reduced sense of stigma [14, 22]

*ART* antiretroviral therapy, *DSD* differentiated service delivery, *HIV* human immune deficiency virus

#### Facility-based individual models

##### Barriers

Inconsistent model implementation [15, 52] and ARV drug stock-outs were organization-related barriers whereas the supply chain inconsistencies [14, 15, 52, 59, 67] were system-related barriers cited by the included studies. Provider-related barriers included a lack of information on model implementation [15], concern about patients’ returning to the clinic to report any problems [14, 52, 55], and fear of missing appointments in multimonth prescriptions [52]. Perceived lack of client-centeredness for the fast track refill model [24] and feasibility issue regarding large volumes of ART drug storage at home for multimonth prescriptions [55] were the patient-related inhibitors for model implementation.

##### Facilitators

From the patients’ side, a perceived higher need for privacy and confidentiality [14, 47, 59, 70], comprehensive health checks before taking necessary medications [49], reduced travel costs [14, 52, 53, 55, 68], reduced waiting time [14, 15, 52, 67, 70] and increased time for income-generating activities [55, 68] were reported as the enablers for model implementation. Reduction in staff workload [15, 47, 52, 53, 55, 67, 68] and decongestion of health facilities with clients [15, 47, 52, 53, 67] were supply-side facilitators commonly reported in the included studies for model implementation.

#### Out of facility-based individual models

##### Barriers

Demand-side barriers included fear of detachment from the health facility [59], patients’ lack of clarity on models [14], fear of missing doses because their medication was not delivered on time at home [15, 61] and fear of accidental disclosure [49]. Supply-side barriers included frequent drug stock-outs and supply chain problems [14], concerns about the need for providers’ monetary allowances and transport costs at communities [59], the difficulty in finding suitable space for outreach ART refills in rural settings [53], and additional burdens of data collection responsibilities [14].

##### Facilitators

From the patients’ side, reduced travel [14, 49], the convenience of accessing medications at home [49], and acceptability of the community pharmacy ART refill model were reported enablers for the implementation of models [44, 63]. Reduction in the overburdening of health facilities with clients [14] and better care for sicker patients [14] were cited as providers-related facilitators*.*

#### Client led group-based models

##### Barriers

Fear of stigma and discrimination for joining groups was a dominant patient-related barrier cited by included studies [14, 50, 59, 62]. Similarly, fear of detachment from the health facility [59], fear of clashing with peers [50], and dissatisfaction with the efficiency of drug pickups at the community level [14] were also patient-related barriers reported by the included studies. Medical record disorganization [14], the additional workload involved in packaging and labeling drugs for each member [59, 70], difficulty in finding competent and literate community client-led model leaders [59], and frequent changes in physical addresses among urban clients [59] were reported barriers for model implementation from the providers’ perspective.

##### Facilitators

From the patients’ side, lower transport costs [15, 56, 59, 62, 70] and increasing group and social support [15, 52, 56] were dominant enablers for model implementation. Reduction in the overburdening of health facilities with clients [15, 56, 62] and more time spent on patient data compilation and viral load testing [15, 62] were cited facilitators in model implementation from the providers’ side.

#### Healthcare worker-led group-based models

##### Barriers

From the patients’ perspective, inadvertent status disclosure [15, 22, 70], infrequent clinician visits [15], needing to find members to join their group [15], challenges with ART supply to adherence clubs [14], and patients’ lack of clarity about how models work [15] were among the barriers cited.

From the providers’ side, the reported barriers include providers’ concerns about insufficient medical recordkeeping [14], incorrect patient differentiation [14, 15], infrastructure (space) concerns [15, 22], and issue of transportation of the prepacked medication to the distribution sites [15, 22], staff shortages [15], and lack of compensation for staff working off-hours [15].

##### Facilitators

Reduced transportation costs [14, 70], promising health outcomes, particularly for patients who work [15], flexibility to pick up ARTs after the appointment date [15], and the possibility of family members picking up ARVs on behalf of patients [14] were the facilitators cited from the patients’ perspective. From the providers’ side, the facilitators reported by the included studies were improvement in adherence to treatment [14, 22], reduction in defaulter rate and tracking of loss to follow up [14, 22], reduction in the overburdening of health facilities with clients [14, 22], and increased opportunities for task-sharing among clinic staff [14, 22].

### Barriers and facilitators common across DSD models implementation

The common reported facilitators for implementing four DSD models include reduced travel cost [14, 15, 49, 52, 53, 55, 56, 59, 62, 68, 70], improved or maintained adherence and retention [14, 15, 22, 56, 63, 67], reduction in staff workload [14, 15, 22, 47, 52, 53, 55, 62, 67, 68], and reduction in the overburdening of health facilities with clients [14, 15, 22, 47, 52, 53, 56, 62, 67].

The fear of stigma and discrimination [14, 15, 22, 47, 49, 50, 59, 62, 70], providers’ concern about patients’ ability to return to the clinic for other illnesses [14, 15, 52, 55, 56] and ARV drug stock-outs and supply chain inconsistencies [14, 15, 52, 59] were the commonly reported barriers for implementing four DSD models (Table 2).

### Barriers and facilitators to the scale-up of DSD models for HIV treatment

Fifteen studies reported on DSD scale up. The barriers and facilitators identified in the included studies were summarized based on the health system dynamics framework in the following section (Table 3).

**Table 3:** Barriers and facilitators to scale-up of differentiated antiretroviral therapy service delivery in Africa, 2021

Factors	Barriers	Facilitators
Client, community, and service provider	▪ Income and residence [59] ▪ Patients’ perception of the terms ‘unstable’ and ‘stable’ in DSD classification as provider stigma [59] ▪ Stigma at the community level [59] ▪ Patients’ low literacy [58, 59] ▪ The lack of buy-in from healthcare workers at both the facility and community levels [35] ▪ The low energy required of providers to initiate or maintain change [38] ▪ The ART program staff’s perception of clubs not being core program work [38] ▪ Healthcare workers’ perception of having an increased workload when scaling up adherence clubs in a facility [38] ▪ Low DSD delivery competence among health workers [59] ▪ Serving patients in community-based models was not seen as the facility’s responsibility [30]	▪ The presence of patient education and peer support [47]
Resources (time, finance, information, space, drug, and workforce)	▪ Inadequate drug supply [38, 47, 59] ▪ Insufficient laboratory testing infrastructure [16] ▪ Complaints about bad infrastructures, such as small rooms and a scarcity of off-site places [38], and no available comfortable seating for adherence club meetings [35] ▪ Financial constraints [12, 25, 40, 54, 59] ▪ In-efficient utilization of existing resources [40] ▪ Inadequate number of staff [38] ▪ Lack of time to allow the client and/or provider buy-in [25]	▪ A consistent and flexible medication supply [45, 47] ▪ The availability of functioning and reliable information system s[45, 47]
Leadership and governance	▪ Weak health system [58] ▪ Lack of effort to formalize plans [38] ▪ Gaps in pharmacy supply chain management [16] ▪ Inadequate forecasting of healthcare worker needs within DSD models [16] ▪ Inadequate training, coordination, and compensation of community healthcare workers [16, 35, 38, 59] ▪ The clash between DSD and tuberculosis appointment spacing [59] ▪ Inconsistency in model uptake and adoption across models [16] ▪ Problems of capacity related to the composition of the chronic dispensing unit system and the pharmaceutical dimension of clubs [38] ▪ DSD not implemented in lower health facilities [59] ▪ DSD lacked client-centeredness as designed [59] ▪ The mix of the adherence club program with other HIV-negative patients [35] ▪ Poor care linkages [47] ▪ Inaccurate differentiating of patients based on clinical stability [16] ▪ Lack of clarity regarding the ongoing role of the steering committee [38]	▪ Political will at all levels of the health system [38] ▪ Policies and guidelines development [47] ▪ Strong care linkages [47] ▪ Clear referral mechanisms between the community and health facility [45] ▪ Provision of free care to access HIV-related services [45] ▪ Availability of central chronic medicine dispensing and distribution program [26] ▪ A sequence of events for stepwise model implementation [57] ▪ Availability of training, strong supervision, and guidance related activities [25, 38, 45, 47] ▪ Remuneration for lay workers involved in supporting community-based models [45] ▪ Availability of a dedicated committee [38] ▪ The better approach of the clubs’ steering committee to guide adherence club eligibility and structure [27] ▪ The collaborative implementation process [12] ▪ Increased focus on person-centered care [25] ▪ The presence of influential people in the steering committee [38] ▪ Deployment of a nurse champion [38] ▪ The influence of early adopter clinics on other clinics providing ART service [38]
Context	▪ Extreme poverty conditions, particularly in rural areas [58] ▪ Frequent changes in physical addresses (mobility) among urban clients [59]	▪ Model flexibility [25]

*ART* antiretroviral therapy, *DSD* differentiated service delivery

#### Population (patient, community, and service provider)

Internalized stigma and discrimination were identified as barriers to scaling up DSD models for HIV treatment [47, 58, 59]. For example, lower-income and rural patients preferred community-based DSD models, whereas urban and wealthier patients preferred facility-based models due to a higher expressed need for privacy and confidentiality [59]. Another barrier reported was patients’ perception of the terms ‘unstable’ and ‘stable’ in DSD classification as provider-initiated stigma [59]. Stigma at the community level was a major impediment to community-based models [59]. Patients’ low literacy level was reported to be a barrier to enrollment in DSD models [58, 59]. The presence of patient education and peer support was found as a facilitator for the scaling up of DSD models [47].

The lack of acceptance from healthcare workers hampered the expansion of the adherence club intervention [35]. Scaling up adherence clubs in a facility was also hampered by the low energy held by providers to initiate or maintain change [38] and the providers’ perception of clubs not being core program work and having an increased workload when scaling up adherence clubs in a facility [38]. Low DSD delivery competence among healthcare workers has been identified as a bottleneck in service expansion [59]. The clinic staff’s low understanding of the benefits of the model and lack of trust that patients could be successfully managed outside of the traditional model of care was a barrier to successful community adherence club scale up [30].

#### Resources (time, finance, information, space, drug, and workforce)

The most common barriers to scaling up DSD models were a lack of financial, human, space, and drug resources as well as a lack of time to allow the client and/or provider buy-in [12, 16, 25, 35, 38, 40, 45, 47, 54, 58, 59]. Inadequate drug supply was reported as a major barrier in the DSD scale-up [38, 47, 59]. A consistent and flexible medication supply, on the other hand, has been found to help the DSD model scale up [45, 47]. Insufficient laboratory testing infrastructure [16], complaints about bad infrastructures such as small rooms and a scarcity of off-site places [38], and no available comfortable seating for adherence club meetings have all been reported as barriers [35]. Financial constraints have emerged as a major barrier to scaling up DSD models [12, 25, 40, 54, 59]. In a similar vein, inefficient utilization of existing resources has been identified as a challenge in model scale up [40]. Inadequate personnel levels have been cited as a barrier to the institutionalization of a pilot innovation [38]. The availability of functioning and reliable information systems aided model scale-up [45, 47].

#### Leadership and governance

The weak health system to maintain community ART group activities [58], lack of effort to formalize plans [38], gaps in pharmacy supply chain management [16], inadequate forecasting of healthcare worker needs within DSD models [16], inadequate training, coordination, and compensation of community healthcare workers [16, 35, 38, 59], and the clash between DSD and tuberculosis appointment spacing [59] were reported as the barriers for model scale up.

Inconsistency in model uptake and adoption [16], DSD not being implemented in lower health facilities [59], and DSD being provider-directed and lacking its client-centered goal [59] were also the reported barriers concerning leadership and governance aspects of DSD scale up efforts. In addition, the mix of the adherence club program with other HIV-negative patients [35], poor care linkages [47], inaccurate differentiating of patients based on clinical stability [16], and lack of clarity regarding the ongoing role of the steering committee [38] were reported barriers to DSD scale up.

In this review, the reported leadership and governance-related facilitators include a sequence of events for stepwise model implementation [57]*,* availability of training, strong supervision and guidance related activities [25, 38, 45, 47], and remuneration for lay workers involved in supporting community-based ART delivery models [45]. Political will at all levels of the health system [38], policies and guidelines development [47], strong care linkages [47], clear referral mechanisms between the community and health facility [45], provision of free care to access HIV-related services [45] and availability of central chronic medicine dispensing and distribution program [26] were identified as critical facilitators.

The availability of a dedicated committee [38], the good approach of the adherence clubs steering committee while supporting individual health facilities offering adherence clubs [27], the collaborative implementation process [12], and increased focus on person-centered care [25] were reported facilitators in the leadership and governance dimension of DSD scale up. In addition, the presence of influential people in the steering committee [38], the deployment of a nurse champion [38], and the influence of early adopter clinics on other clinics providing ART service [38] were also reported as the leadership and governance related facilitators in model scale up.

#### Context

Extreme poverty, particularly in rural areas, was a barrier to the institutionalization of community-based models [58]. The running of patient groups of rotating ART refill pick-ups has been reported to be hampered by frequent changes in physical addresses (mobility) among urban clients [59]. Model flexibility was reported as the facilitator for scaling up the DSD models [25].

## Discussion

### Summary of the main results

Our scoping review aimed to identify the barriers and facilitators that influence the implementation and scale up of DSD interventions. The review identified several barriers and facilitators related to DSD model implementation and scale up. The synthesis showed that the overall influencing factors were clustered based on the four major types of DSD models for implementation, and according to the health system dynamics framework for scale up.

### Implementation of DSD interventions

In this review, multiple barriers and facilitators were reported in the implementation of DSD models from both patient and provider perspectives. There is inconsistency in the influencing factors across the DSD models. This most likely reflects the differing circumstances and the effectiveness with which models were implemented as well as the inherent characteristics of each respective model. This is in agreement with a previous study which identified that different models place different demands on the health system and employ different techniques to break down barriers to care, therefore their functions may vary depending on the situation [73]. This has important implications for further policy development across health systems to accelerate the adaptation of DSD models in each setting.

The most often stated challenges to model implementation were staff shortages, providers’ lack of information on model implementation, and lack of staff clarity on eligibility criteria. These have policy implications to avail sufficient numbers and a diverse range of DSD workers, who are given the necessary training, skills, and tool to ensure that DSD is implemented with competence, responsiveness, and productivity. Low patient literacy and a lack of understanding of how the models work were also barriers to model implementation. This implies that extensive, comprehensive, and ongoing patient counseling and health promotion on DSD models are needed.

The availability of low or declining funding to support DSD models as well as limited logistics such as insufficient drug supply, lack of space for group-based models and lack of transportation of the prepacked medication to the distribution sites have created pressure for the adoption and implementation of HIV treatment models as reported from both patient and provider perspectives. This is consistent with a previous review [16].

The issues of stigma and discrimination were paramount concerns raised by patients which affect the implementation of respective DSD models. A previous study also identified stigma and discrimination as a barrier to ending AIDS by 2030 and achieving the 90-90-90 targets by 2020 [74]. This highlights much effort is needed to achieve the UNAIDS’s vision of zero discrimination toward people living with HIV by 2030.

Reduced patient travel costs, reduction in staff workload, and reduction in the overburdening of health facilities with clients and hence higher quality of care for unstable patients as well as improved or maintained adherence and retention were common reported facilitators for implementing DSD models for HIV treatment. These have important implications for health system performance (access, coverage, efficiency, equity, quality, safety, and sustainability) and overall impact (improved health, risk protection, and responsiveness) [75].

### Scale-up of DSD interventions

The weak health systems, leadership, and governance were often reported as a barrier to DSD scale up. This could restrict the path to the Universal Health Care goal to be achieved by 2030. Moving closer to this goal requires the needed health services (such as DSD) to be available, of good quality, and affordable, which in turn requires attention to all the various components of a health system (infrastructure, medicines and medical products, health workers, health information, and health system financing). In this regard, good leadership and governance are critical and relevant to all the health system components as well as to the interactions between them [76]. A previous systematic review in sub-Saharan Africa also identified that a clear vision for institutionalizing DSD, innovative monitoring, and capacitating the health system with basic human and material resources are required to facilitate DSD sustainability [77]. Continuation of the existing weak health system, leadership, and governance however might impede the progress toward the next DSD 2.0 model, which integrates ART services with the most common vertical programs that require repeated follow-up: Tuberculosis prevention and treatment, family planning, and chronic non-communicable diseases as emphasized by WHO’s 2016 HIV guidelines [3].

The other most prominent factor influencing the scale-up of DSD interventions was the availability of the related resources. Lack of financial, human, space, and drug resources, as well as a lack of time to allow the client and/or provider buy-in, were often barriers to scale up. This could be detrimental to achieving the aim of DSD. According to the WHO’s consolidated guidelines on HIV prevention, testing, treatment, service delivery, and monitoring the success of DSD models in delivering ART depends on sufficient, reliable support and resources, such as a cadre of trained lay workers, a flexible and reliable medication supply, access to quality clinical management, and a reliable monitoring system for comprehensive client care [5].

The other common barriers to scaling up DSD models were the internalized stigma and discrimination issues attached to varied HIV treatment models. These two twin barriers might lead to a delay in the UNAIDS’ global partnership’s goal to reach zero HIV-related stigma and discrimination by 2030 [78].

Political will, policies and guidelines development, strong care linkages, clear referral mechanisms between the community, and health facility and provision of free care to access HIV-related services were facilitators for DSD model scale up. This has important implications for policymakers, program managers, and practitioners to enhance the existing leadership and governance efforts for continued expansion and maintenance of DSD models.

### Strengths and limitations of the review

We emphasize that the strength of this review lies in drawing quantitative, qualitative, and mixed methods studies from a variety of evidence sources in a way not done previously, and we believe that our review still adds value to the current body of knowledge on DSD, by providing collated and comprehensive insights into the peer-reviewed scientific literature. The inclusion of grey literature makes this scoping review novel within this topic since the previous reviews were limited to published articles where valuable information from grey literature might have been overlooked. It is also a strength of this review that we have reviewed the perspectives of both patients and service providers that have not been adequately researched to date.

This review has limitations that should be taken into consideration when interpreting the results. First, the specific objective of all of the studies included was not the identification of barriers or enablers to DSD model implementation. The inconsistency noted was therefore expected, as barriers and enablers had to be extracted from the study reports, as thematic outputs. Second, as with the limitations of any scoping review, there is the possibility of incomplete retrieval of identified research due to the scope of the search terms and the databases searched. Third, there might be a probability of selection bias as only studies in the English language were included. Fourth, as this was a scoping review, we also did not perform a quality assessment therefore implications for practice or policy cannot be graded. Fifth, generalization of the study findings to settings other than Africa could be difficult due to variations in health systems and resource availability. It could even be difficult to generalize study findings to some settings in Africa since the continent encompasses a vast range of cultures and communities.

## Conclusions

This scoping review identified a broad range of factors across multiple levels affecting the implementation and scale-up of different alternative DSD innovative interventions. There was an inconsistency in reporting factors by the included studies in this review where the same factor might be a facilitator in one context and a barrier in another context. The findings provide preliminary information to practitioners, program managers, decision makers, policymakers, educators, and researchers involved in the planning, design, implementation, scale-up, and evaluation of DSD models for HIV treatment. However, a major knowledge gap remains when it comes to understanding which contextual factors influence DSD implementation and scale-up in each setting. Hence, large-scale studies informed by implementation and scale up theories, models, and frameworks focusing on each DSD model in each healthcare setting are needed. In addition, there is a need for studies that explore the interrelationships between the various levels of barriers and facilitators identified in this review. Another unanswered question is related to the relative importance of each factor in specific DSD model implementation and scale up contexts which need to be explored by studies using prospective designs.

## Supplementary Information

**Additional file 1. **Search strategy details.

**Additional file 2.**

## Data Availability

All data generated or analysed during this study are included in this published article and its supplementary information files.

## Abbreviations

AIDS: Acquired Immunodeficiency Syndrome
ART: Antiretroviral Therapy
ARV: Antiretroviral
DSD: Differentiated Service Delivery
HIV: Human Immunodeficiency Virus
JBI: Jonna Briggs Institute
PRISMA: Preferred Reporting Items for Systematic Reviews and Meta-Analyses
UNAIDS: The Joint United Nations Programme on HIV/AIDS
WHO: World Health Organization

## References

[1] HIV/AIDS _Fact Sheet. Available from: https://www.who.int/news-room/fact-sheets/detail/hiv-aid. Accessed 31 Dec 2020.

[2] Joint United Nations Programme on HIV/AIDS (2014). 90-90-90: an ambitious treatment target to help end the AIDS epidemic.

[3] World Health Organization (2016). Consolidated guidelines on the use of antiretroviral drugs for treating and preventing HIV infection: recommendations for a public health approach.

[4] Duncombe C, Rosenblum S, Hellmann N, Holmes C, Wilkinson L, Biot M, Bygrave H, Hoos D, Garnett GJTM (2015). Health I: reframing HIV care: putting people at the centre of antiretroviral delivery. Tropical Med Int Health.

[5] World Health Organization (2021). Consolidated guidelines on HIV prevention, testing, treatment, service delivery and monitoring: recommendations for a public health approach.

[6] International AIDS Society (2022). Differentiated care models.

[7] El-Sadr W (2018). Differentiated service delivery: taking innovative delivery models to scale.

[8] Ford N, Geng E, Ellman T, Orrell C, Ehrenkranz P, Sikazwe I, Jahn A, Rabkin M, Ayisi Addo S, Grimsrud AJ (2020). Emerging priorities for HIV service delivery. PLoS Med.

[9] Ehrenkranz P, Grimsrud A, M R (2019). AIDS: differentiated service delivery: navigating the path to scale. Curr Opin HIV AIDS.

[10] Glasgow RE, Vinson C, Chambers D, Khoury MJ, Kaplan RM, Hunter CJ (2012). National institutes of health approaches to dissemination and implementation science: current and future directions. Am J Public Health.

[11] Organization WH (2010). Nine steps for developing a scaling-up strategy.

[12] Flamig K, Decroo T, van den Borne B, van de Pas R (2019). ART adherence clubs in the Western cape of South Africa: what does the sustainability framework tell us? A scoping literature review. J Int AIDS Soc.

[13] Hagey JM, Li X, Barr-Walker J, Penner J, Kadima J, Oyaro P, Cohen CR (2018). Differentiated HIV care in sub-Saharan Africa: a scoping review to inform antiretroviral therapy provision for stable HIV-infected individuals in Kenya. AIDS Care.

[14] Kuchukhidze S, Long L, Pascoe S, Huber A, Nichols B, Fox M. Differentiated models of service delivery (DSD) for antiretroviral treatment of HIV in sub-Saharan Africa: a review of the gray literature as of June 2019. In: AMBIT Project Report. 10.1186/s13643-019-1210-6 PMC6896778 31810482

[15] Long L, Kuchukhidze S, Pascoe S, Nichols B, Cele R, Govathson C (2020). Differentiated service delivery models for antiretroviral treatment of HIV in Sub-Saharan Africa: a rapid systematic review.

[16] Roy M, Bolton Moore C, Sikazwe I, Holmes CB (2019). A review of differentiated service delivery for HIV treatment: effectiveness, mechanisms, targeting, and scale. Curr HIV/AIDS Rep.

[17] Peters MDJGC, McInerney P, Munn Z, Tricco AC, Khalil H (2020). JBI manual for evidence synthesis. Chapter 11: scoping reviews.

[18] Tricco AC, Lillie E, Zarin W, O'Brien KK, Colquhoun H, Levac D, Moher D, Peters MD, Horsley T, Weeks LJ (2018). PRISMA extension for scoping reviews (PRISMA-ScR): checklist and explanation. Ann Intern Med.

[19] JBI Manual for Evidence Synthesis. https://wiki.joannabriggs.org/display/MANUAL.

[20] van Olmen J, Criel B, Van Damme W, Marchal B, Van Belle S, Van Dormael M, Hoerée T, Pirard M, Kegels G (2010). Analysing health systems to make them stronger.

[21] Peters M, Godfrey C, McInerney P, Soares C, Khalil H, Parker D (2015). The Joanna Briggs institute reviewers’ manual 2015: methodology for JBI scoping reviews.

[22] Tshuma N, Mosikare O, Yun JA, Alaba OA, Maheedhariah MS, Muloongo K, Nyasulu PS (2017). Adherence: acceptability of community-based adherence clubs among health facility staff in South Africa: a qualitative study. Patient Prefer Adherence.

[23] Mukumbang FC, Bv W, Sv B, Marchal B (2019). 'At this [adherence] club, we are a family now': a realist theory-testing case study of the antiretroviral treatment adherence club, South Africa. Southern Afr J HIV Med.

[24] Ndlovu S (2020). Comparison of patient experiences in three differentiated antiretroviral delivery models in a public health care facility.

[25] Sharer M, Davis N, Makina N, Duffy M, Eagan S (2019). Differentiated antiretroviral therapy delivery: implementation barriers and enablers in South Africa. J Assoc Nurses AIDS Care.

[26] Liu L, Christie S, Munsamy M, Roberts P, Pillay M, Shenoi S, Desai M, Linnander E (2021). Expansion of a national differentiated service delivery model increases access to treatment for HIV and other chronic conditions in South Africa.

[27] Wilkinson L, Harley B, Sharp J, Solomon S, Jacobs S, Cragg C, Kriel E, Peton N, Jennings K, Grimsrud A (2016). Expansion of the adherence Club model for stable antiretroviral therapy patients in the cape metro, South Africa 2011-2015. Tropical Med Int Health.

[28] Dudhia R, AJP K (2015). Experiences of participating in an antiretroviral treatment adherence club. Psychol Health Med.

[29] Venables E, Towriss C, Rini Z, Nxiba X, Solomon S, Cassidy T, et al. If I’m not in the club, I have to move from one chair to another. In: A qualitative evaluation of patient experiences of adherence clubs in Khayelitsha and Gugulethu, South Africa 9th IAS Conf HIV Sci. Paris; 2017.

[30] Grimsrud A, Sharp J, Kalombo C, Bekker LG, Myer L (2015). Implementation of community-based adherence clubs for stable antiretroviral therapy patients in Cape Town, South Africa. J Int AIDS Soc.

[31] Wilkinson L, Harley B, Jacobs S, Cragg C, Kriel E, Solomon S, Peton N, Jennings K, Youngleson M, Grimsrud A (2015). Implementation scale up of the adherence Club model of care to 30,000 stable antiretroviral therapy patients in the cape metro: 2011-2014. J Int AIDS Soc.

[32] Keene CM, Zokufa N, Venables EC, Wilkinson L, Hoffman R, Cassidy T, Snyman L, Grimsrud A, Voget J, von der Heyden EJ (2020). Only twice a year’: a qualitative exploration of 6-month antiretroviral treatment refills in adherence clubs for people living with HIV in Khayelitsha, South Africa. BMJ Open.

[33] Venables E, Towriss C, Rini Z, Nxiba X, Cassidy T, Tutu S, Grimsrud A, Myer L, Wilkinson L (2019). Patient experiences of ART adherence clubs in Khayelitsha and Gugulethu, Cape Town, South Africa: a qualitative study. PLoS One.

[34] De Jager GA, Crowley T, Esterhuizen TM (2018). Patient satisfaction and treatment adherence of stable human immunodeficiency virus-positive patients in antiretroviral adherence clubs and clinics. Afr J Prim Health Care Fam Med.

[35] Mukumbang FC, Marchal B, Van Belle S, van Wyk B (2018). "Patients are not following the [adherence] Club rules anymore": a realist case study of the antiretroviral treatment adherence Club, South Africa. Qual Health Res.

[36] Mudavanhu M, West NS, Schwartz SR, Mutunga L, Keyser V, Bassett J, Van Rie A, Hanrahan CF (2020). Perceptions of community and clinic-based adherence clubs for patients stable on antiretroviral treatment: a mixed methods study. AIDS Behav.

[37] Bock P, Gunst C, Maschilla L, Holtman R, Grobbelaar N, Wademan D, et al. Retention in care and factors critical for effectively implementing antiretroviral adherence clubs in a rural district in South Africa. J Int AIDS Soc. 2019;22(10). 10.1002/jia2.25396 PMC6778813 31588668

[38] MacGregor H, McKenzie A, Jacobs T, Ullauri AJG (2018). Health: scaling up ART adherence clubs in the public sector health system in the Western cape, South Africa: a study of the institutionalisation of a pilot innovation. Glob Health.

[39] Dorward J, Msimango L, Gibbs A, Shozi H, Tonkin-Crine S, Hayward G, Butler CC, Ngobese H, Drain PK, Garrett N (2020). Understanding how community antiretroviral delivery influences engagement in HIV care: a qualitative assessment of the centralised chronic medication dispensing and distribution programme in South Africa. BMJ Open.

[40] Mukumbang FC, Orth Z, Van Wyk B (2019). Aystems: what do the implementation outcome variables tell us about the scaling-up of the antiretroviral treatment adherence clubs in South Africa? A document review. Health Res Policy Syst.

[41] Davey D, Serrao C, Prins M, Feinberg M, Pooe D, Femi O (2018). Who were the patients on differentiated care? Uptake of HIV differentiated care models for patients on antiretroviral therapy in South Africa. Abstract WEPEE756. 22nd international AIDS conference, Amsterdam.

[42] Laga M: Community-based ART in sub-Saharan Africa: lessons learnt from community ART groups in Tete province, Mozambique.

[43] Rasschaert F, Decroo T, Remartinez D, Telfer B, Lessitala F, Biot M, Candrinho B, Van Damme W (2014). Adapting a community-based ART delivery model to the patients’ needs: a mixed methods research in Tete, Mozambique. BMC Public Health.

[44] Asieba IO, Oqua DA, Wutoh AA, Agu KA, Omeh OI, Adeyanju ZA, Adesina A, Agu F, Agada P, Achanya A (2021). Antiretroviral therapy in community pharmacies - implementation and outcomes of a differentiated drug delivery model in Nigeria. Res Soc Adm Pharm.

[45] Bemelmans M, Baert S, Goemaere E, Wilkinson L, Vandendyck M, van Cutsem G, Silva C, Perry S, Szumilin E, Gerstenhaber RJTM (2014). Community-supported models of care for people on HIV treatment in sub-S aharan A frica. Tropical Med Int Health.

[46] Ssonko C, Gonzalez L, Mesic A, da Fonseca MS, Achar J, Safar N, Martin B, Wong S, Casas EC (2017). Delivering HIV care in challenging operating environments: the MSF experience towards differentiated models of care for settings with multiple basic health care needs. J Int AIDS Soc.

[47] Duffy M, Sharer M, Davis N, Eagan S, Haruzivishe C, Katana M, Makina N, Amanyeiwe U (2019). Differentiated antiretroviral therapy distribution models: enablers and barriers to universal HIV treatment in South Africa, Uganda, and Zimbabwe. J Assoc Nurses AIDS Care.

[48] Huber A, Pascoe S, Nichols BE, Long L, Kuchukhidze S, Phiri B, Tchereni T, S R (2020). Sifferentiated service delivery models for HIV treatment in Malawi, South Africa, and Zambia: a landscape analysis.

[49] Adjetey V, Obiri-Yeboah D, B D (2019). Differentiated service delivery: a qualitative study of people living with HIV and accessing care in a tertiary facility in Ghana. BMC Health Serv Res.

[50] Kizito O, Sabiti LJCM (2021). Factors associated with uptake of community client-led ART delivery model at Mulago adult HIV clinic _ Mulago National Referral Hospital. Cogent Med.

[51] Zakumumpa H, Bennett S, Ssengooba F (2017). Modifications to ART service delivery models by health facilities in Uganda in promotion of intervention sustainability: a mixed methods study. Implement Sci.

[52] Prust ML, Banda CK, Callahan K, Nyirenda R, Chimbwandira F, Kalua T, Eliya M, Ehrenkranz P, Prescott M, E MC (2018). Patient and health worker experiences of differentiated models of care for stable HIV patients in Malawi: a qualitative study. PLoS One.

[53] Phiri K, McBride K, Siwale Z, Hubbard J, Bardon A, Moucheraud C, Haambokoma M, Pisa PT, Moyo C, RM H (2021). Provider experiences with three-and six-month antiretroviral therapy dispensing for stable clients in Zambia. AIDS Care.

[54] Rasschaert F, Telfer B, Lessitala F, Decroo T, Remartinez D, Biot M, Candrinho B, Mbofana F, Van Damme W (2014). A qualitative assessment of a community antiretroviral therapy group model in Tete, Mozambique. PLoS One.

[55] Hubbard J, Phiri K, Moucheraud C, McBride K, Bardon A, Balakasi K, Lungu E, Dovel K, Kakwesa G, Hoffman RM (2020). A qualitative assessment of provider and client experiences with 3-and 6-month dispensing intervals of antiretroviral therapy in Malawi. Glob Health Sci Pract.

[56] Bochner AF, Meacham E, Mhungu N, Manyanga P, Petracca F, Muserere C, Gonese G, Makunike B, Wazara B, Gwanzura CJ (2019). The rollout of community ART refill groups in Zimbabwe: a qualitative evaluation. J Int AIDS Soc.

[57] Decroo T, Lara J, Rasschaert F, Bermudez-Aza E, Couto A, Candrinho B, et al. Scaling up community ART groups in Mozambique. Int STD Res Rev. 2013;12:49–59.

[58] Rasschaert F, Decroo T, Remartinez D, Telfer B, Lessitala F, Biot M, Candrinho B, Van Damme W (2014). Sustainability of a community-based anti-retroviral care delivery model - a qualitative research study in Tete, Mozambique. J Int AIDS Soc.

[59] Zakumumpa H, Rujumba J, Kwiringira J, Katureebe C, Spicer N (2020). Understanding implementation barriers in the national scale-up of differentiated ART delivery in Uganda. BMC Health Serv Res.

[60] Pellecchia U, Baert S, Nundwe S, Bwanali A, Zamadenga B, Metcalf CA, Bygrave H, Daho S, Ohler L, Chibwandira B (2017). “We are part of a family” Benefits and limitations of community ART groups (CAGs) in Thyolo, Malawi: a qualitative study. J Int AIDS Soc.

[61] Geldsetzer P, Francis JM, Ulenga N, Sando D, Lema I, Mboggo E, Vaikath M, Lwezaula S, Koda H, Hu J, Noor R, Olofin I, Fawzi W, Bärnighausen T (2017). The acceptability and feasibility of community health worker-led home-delivery of antiretroviral therapy: early findings from a health systems trial in Dar es Salaam, Tanzania.

[62] Mashungu V, Munkuli J, Khembo W, Kumani B, Nkawu B, Fatti G. Acceptability of community differentiated models of care: a patient and service provider’s perspective on community art refill groups in Zimbabwe. Abstract WEPEE759. In: 22nd international AIDS conference. Amsterdam; 2018.

[63] Mulenga HB, Muhau M, Kateule K. Differentiated service delivery using a “community pharmacy ART drug pickup” model: an evaluation from Lusaka, Zambia**.** Abstract MOPED623. In: 10th IAS conference on HIV science. Mexico City; 2019.

[64] Roy M, Bolton C, Sikazwe I, Mukumbwa-Mwenechanya M, Efronson E, Somwe P. Implementation and effectiveness of urban adherence clubs in Zambia. Abstract FRAE0104. In: 22nd international AIDS conference. Amsterdam; 2018.

[65] Pasipamire L, Kerschberger B, Zabsonre I, Ndlovu S, Sibanda G, Mamba S, Mazibuko S, Lukhele N, Kabore S, Rusch B (2016). Implementation of combination ART refills models in rural Swaziland. Journal of THE international AIDS Society.

[66] Prust M, Banda C, Nyirenda R, Chimbwandira F, Kalua T, Jahn A, Eliya M, Callahan K, Ehrenkranz P, Prescott M (2017). Multi-month refills of antiretroviral drugs for stable patients in Malawi: assessing accuracy in the application of eligibility criteria at the health facility level. Journal Of the international aids society.

[67] Attah M, Mohammed A, Hassan S. Nigeria ARV multimonth scripting: impact on public health service delivery, infrastructure and supply chain management systems across “high volume” ART clinics. Abstract WEPEE740. In: 22nd international AIDS conference. Amsterdam; 2018.

[68] Hubbard J, Phiri K, Moucheraud C, McBride K, Bardon A, Balakasi K, Lungu E, Dovel K, Kakwesa G, Hoffman RM: Understanding implementation from provider and client perspectives: The INTERVAL study on multi-month ART dispensing in Malawi.

[69] Roy M, Mukumbwa-Mwenechanya M, Efronson E, Lumpa M, Sharma A, Sikazwe I, et al. Uptake and adaptation of community adherence groups in Zambia. In: Poster presentation at the conference on retroviruses and opportunistic infections. Seattle; 2017. p. 13–6.

[70] Christ B, van Dijk JH, Ballif M, Nyandoro T, Reichmuth ML, Mukondwa WR, Kunzekwenyika C, Manhibi R, Tasunga D, Chammartin F (2020). Differentiated antiretroviral therapy delivery in rural Zimbabwe: availability, needs and challenges.

[71] Zakumumpa H, Makobu K, Wilbrod N, Maniple E (2021). A mixed-methods evaluation of the uptake of novel differentiated ART delivery models in a national sample of health facilities in Uganda.

[72] Prust ML, Banda CK, Nyirenda R, Chimbwandira F, Kalua T, Jahn A, Eliya M, Callahan K, Ehrenkranz P, Prescott MR (2017). Multi-month prescriptions, fast-track refills, and community ART groups: results from a process evaluation in Malawi on using differentiated models of care to achieve national HIV treatment goals. J Int AIDS Soc.

[73] Geng EH, Holmes CB (2019). Research to improve differentiated HIV service delivery interventions: Learning to learn as we do. PLoS Med.

[74] Wirawan DN (2019). Stigma and discrimination: barrier for ending AIDS by 2030 and achieving the 90-90-90 targets by 2020.

[75] Organization WH (2010). Monitoring the building blocks of health systems: a handbook of indicators and their measurement strategies.

[76] World Health Organization (2014). Health systems governance for universal health coverage action plan: department of health systems governance and financing.

[77] Okere NE, Lennox L, Urlings L, Ford N, Naniche D, de Wit TFR, Hermans S, Gomez GB (2021). Exploring sustainability in the era of differentiated HIV service delivery in Sub-Saharan Africa: a systematic review. J Acquir Immune Defic Syndr.

[78] Global Partnership for Action to Eliminate All Forms of HIV-Related Stigma and Discrimination**.** https://www.unaids.org/en/resources/documents/2018/global-partnership-hiv-stigma-discrimination.

