# Supplementary material for: Barriers and facilitators to the implementation and scale up of differentiated service delivery models for HIV treatment in Africa: a scoping review
Source: BMC Health Serv Res. 2022 Nov 28;22:1431. doi: 10.1186/s12913-022-08825-2 (PMC9703668; doi:10.1186/s12913-022-08825-2)
Supplement: Supplementary file 1 — Additional file 1. Search strategy details. [file 12913_2022_8825_MOESM1_ESM.docx]

**Additional file1: Search strategy details**

| Database | Search Strategy | Number of Results |
| --- | --- | --- |
| PubMed | (patient* OR client* OR provider*) AND ("human immunodeficiency virus" OR "human immunodeficiency virus infection" OR HIV OR "antiretroviral treatment" OR "antiretroviral therapy" OR "antiretroviral therapy, highly active" OR "highly active antiretroviral therapy" OR HAART OR ART) AND ("patient-centred care" OR "patient-centered care" OR "community supported models" OR "adherence club*" OR "task shifting" OR "community ART distribution" OR "community ART delivery" OR "community ART refill" OR "community client lead ART-delivery" OR "facility fast track" OR "quick pick-up" OR "differentiated care" OR "differentiated service" OR "differentiated intervention" OR "decentrali?ed care" OR "decentrali?ed service" OR "decentrali?ed intervention" OR "community care" OR "community service" OR "community intervention" OR "differentiated model*" OR down-referr* OR out-of-clinic) AND (experience* OR attitude* OR perception* OR learning OR Barrie* OR challeng* OR facilitator* OR enabler* OR benefit* OR success* OR constrain* OR difficult* OR enhanc* OR influen* OR interfer* OR motivat* OR obstruct* OR problem* OR promot* OR restrain* OR restrict* OR implement* OR uptake OR adopt* OR adapt* OR accept* OR react* OR appropr* OR feasib* OR fidelity OR sustain* OR modification OR scale-up OR scaling-up OR scale up OR scale-out OR expan* OR replica* OR exten* OR institutionali?ation OR maintain OR continue*) AND (africa OR nigeria OR ethiopia OR egypt OR  "DR congo" OR tanzania OR "south africa" OR kenya OR uganda OR algeria OR sudan OR morocco OR angola OR mozambique OR ghana OR madagascar OR cameroon OR "côte d'Ivoire" OR niger OR "burkina faso" OR mali OR malawi OR zambia OR Senegal OR chad OR Somalia OR zimbabwe OR guinea OR rwanda OR benin OR burundi OR tunisia OR "south sudan" OR  togo OR "sierra leone" OR libya OR congo OR liberia OR "central african republic" OR mauritania OR eritrea OR namibia OR gambia OR  botswana OR gabon OR lesotho OR "guinea-bissau" OR "equatorial guinea" OR mauritius OR eswatini OR djibouti OR comoros OR "cabo verde" OR "sao tome" OR seychelles) NOT (pmtct OR mtct OR mother OR pregnant OR pregnancy OR lactating OR children OR adolescents OR "people who inject drugs" OR MSM OR "transgender persons" OR "sex workers" OR prisoners OR migrants) | 1,045 |
| Scopus | ALL ( patient* OR client* OR provider* ) AND ALL ( "human immunodeficiency virus" OR "human immunodeficiency virus infection" OR hiv OR "antiretroviral treatment" OR "antiretroviral therapy" OR "antiretroviral therapy, highly active" OR "highly active antiretroviral therapy" OR haart OR art ) AND ALL ( "patient-centred care" OR "patient-centered care" OR "community supported models" OR "adherence club*" OR "task shifting" OR "community ART distribution" OR "community ART delivery" OR "community ART refill" OR "community client lead ART-delivery" OR "facility fast track" OR "quick pick-up" OR "differentiated care" OR "differentiated service" OR "differentiated intervention" OR "decentrali?ed care" OR "decentrali?ed service" OR "decentrali?ed intervention" OR "community care" OR "community service" OR "community intervention" OR "differentiated model*" OR down-referr* OR out-of-clinic ) AND ALL ( experience* OR attitude* OR perception* OR learning OR barrie* OR challeng* OR facilitator* OR enabler* OR benefit* OR success* OR constrain* OR difficult* OR enhanc* OR influen* OR interfer* OR motivat* OR obstruct* OR problem* OR promot* OR restrain* OR restrict* OR implement* OR uptake OR adopt* OR adapt* OR accept* OR react* OR appropr* OR feasib* OR fidelity OR sustain* OR modification OR scale-up OR scaling-up OR scale AND up OR scale-out OR expan* OR replica* OR exten* OR institutionali?ation OR maintain OR continue* ) AND ALL ( africa OR nigeria OR ethiopia OR egypt OR "DR congo" OR tanzania OR "south africa" OR kenya OR uganda OR algeria OR sudan OR morocco OR angola OR mozambique OR ghana OR madagascar OR cameroon OR "côte d'Ivoire" OR niger OR "burkina faso" OR mali OR malawi OR zambia OR senegal OR chad OR somalia OR zimbabwe OR guinea OR rwanda OR benin OR burundi OR tunisia OR "south sudan" OR togo OR "sierra leone" OR libya OR congo OR liberia OR "central african republic" OR mauritania OR eritrea OR namibia OR gambia OR botswana OR gabon OR lesotho OR "guinea-bissau" OR "equatorial guinea" OR mauritius OR eswatini OR djibouti OR comoros OR "cabo verde" OR "sao tome" OR seychelles ) AND NOT ALL ( pmtct OR mtct OR mother OR pregnant OR pregnancy OR lactating OR children OR adolescents OR "people who inject drugs" OR msm OR "transgender persons" OR "sex workers" OR prisoners OR migrants ) AND ( LIMIT-TO ( LANGUAGE , "English" ) ) | 1,013 |
| ***Embase*** | (patient* OR client* OR provider*) AND ('human immunodeficiency virus'/exp OR 'human immunodeficiency virus' OR 'human immunodeficiency virus infection'/exp OR 'human immunodeficiency virus infection' OR 'hiv'/exp OR hiv OR 'antiretroviral treatment'/exp OR 'antiretroviral treatment' OR 'antiretroviral therapy'/exp OR 'antiretroviral therapy' OR 'antiretroviral therapy, highly active'/exp OR 'antiretroviral therapy, highly active' OR 'highly active antiretroviral therapy'/exp OR 'highly active antiretroviral therapy' OR 'haart'/exp OR haart OR 'art'/exp OR art) AND ('patient-centred care' OR 'patient-centered care'/exp OR 'patient-centered care' OR 'community supported models' OR 'adherence club*' OR 'task shifting'/exp OR 'task shifting' OR 'community art distribution' OR 'community art delivery' OR 'community art refill' OR 'community client lead art-delivery' OR 'facility fast track' OR 'quick pick-up' OR 'differentiated care' OR 'differentiated service' OR 'differentiated intervention' OR 'decentrali?ed care' OR 'decentrali?ed service' OR 'decentrali?ed intervention' OR 'community care'/exp OR 'community care' OR 'community service'/exp OR 'community service' OR 'community intervention'/exp OR 'community intervention' OR 'differentiated model*' OR 'down referr*' OR 'out of clinic') AND ((experience* OR attitude* OR perception* OR 'learning'/exp OR learning OR barrie* OR challeng* OR facilitator* OR enabler* OR benefit* OR success* OR constrain* OR difficult* OR enhanc* OR influen* OR interfer* OR motivat* OR obstruct* OR problem* OR promot* OR restrain* OR restrict* OR implement* OR uptake OR adopt* OR adapt* OR accept* OR react* OR appropr* OR feasib* OR 'fidelity'/exp OR fidelity OR sustain* OR 'modification'/exp OR modification OR 'scale up'/exp OR 'scale up' OR 'scaling up'/exp OR 'scaling up' OR 'scale'/exp OR scale) AND up OR 'scale out' OR expan* OR replica* OR exten* OR institutionali?ation OR maintain OR continue*) AND ('africa'/exp OR africa OR 'nigeria'/exp OR nigeria OR 'ethiopia'/exp OR ethiopia OR 'egypt'/exp OR egypt OR 'dr congo' OR 'tanzania'/exp OR tanzania OR 'south africa'/exp OR 'south africa' OR 'kenya'/exp OR kenya OR 'uganda'/exp OR uganda OR 'algeria'/exp OR algeria OR 'sudan'/exp OR sudan OR 'morocco'/exp OR morocco OR 'angola'/exp OR angola OR 'mozambique'/exp OR mozambique OR 'ghana'/exp OR ghana OR 'madagascar'/exp OR madagascar OR 'cameroon'/exp OR cameroon OR 'ivory'/exp OR ivory OR 'niger'/exp OR niger OR 'burkina faso'/exp OR 'burkina faso' OR 'mali'/exp OR mali OR 'malawi'/exp OR malawi OR 'zambia'/exp OR zambia OR 'senegal'/exp OR senegal OR 'chad'/exp OR chad OR 'somalia'/exp OR somalia OR 'zimbabwe'/exp OR zimbabwe OR 'guinea'/exp OR guinea OR 'rwanda'/exp OR rwanda OR 'benin'/exp OR benin OR 'burundi'/exp OR burundi OR 'tunisia'/exp OR tunisia OR 'south sudan'/exp OR 'south sudan' OR 'togo'/exp OR togo OR 'sierra leone'/exp OR 'sierra leone' OR 'libya'/exp OR libya OR 'congo'/exp OR congo OR 'liberia'/exp OR liberia OR 'central african republic'/exp OR 'central african republic' OR 'mauritania'/exp OR mauritania OR 'eritrea'/exp OR eritrea OR 'namibia'/exp OR namibia OR 'gambia'/exp OR gambia OR 'botswana'/exp OR botswana OR 'gabon'/exp OR gabon OR 'lesotho'/exp OR lesotho OR 'guinea-bissau'/exp OR 'guinea-bissau' OR 'equatorial guinea'/exp OR 'equatorial guinea' OR 'mauritius'/exp OR mauritius OR 'eswatini'/exp OR eswatini OR 'djibouti'/exp OR djibouti OR 'comoros'/exp OR comoros OR 'cabo verde'/exp OR 'cabo verde' OR 'sao tome' OR 'seychelles'/exp OR seychelles) NOT (pmtct OR mtct OR 'mother'/exp OR mother OR pregnant OR 'pregnancy'/exp OR pregnancy OR lactating OR 'children'/exp OR children OR 'adolescents'/exp OR adolescents OR 'people who inject drugs'/exp OR 'people who inject drugs' OR msm OR 'transgender persons'/exp OR 'transgender persons' OR 'sex workers'/exp OR 'sex workers' OR 'prisoners'/exp OR prisoners OR 'migrants'/exp OR migrants) | 1,442 |
| **Web of Science Core Collection** | TS=(patient* OR client* OR provider*) AND TS= ("human immunodeficiency virus" OR "human immunodeficiency virus infection" OR HIV OR "antiretroviral treatment" OR "antiretroviral therapy" OR "antiretroviral therapy, highly active" OR "highly active antiretroviral therapy" OR HAART OR ART) AND TS=("patient-centred care" OR "patient-centered care" OR "community supported models" OR "adherence club*" OR "task shifting" OR "community ART distribution" OR "community ART delivery" OR "community ART refill" OR "community client lead ART-delivery" OR "facility fast track" OR "quick pick-up" OR "differentiated care" OR "differentiated service" OR "differentiated intervention" OR "decentrali?ed care" OR "decentrali?ed service" OR "decentrali?ed intervention" OR "community care" OR "community service" OR "community intervention" OR "differentiated model*" OR down-referr* OR out-of-clinic) AND TS= (experience* OR attitude* OR perception* OR learning OR Barrie* OR challeng* OR facilitator* OR enabler* OR benefit* OR success* OR constrain* OR difficult* OR enhanc* OR influen* OR interfer* OR motivat* OR obstruct* OR problem* OR promot* OR restrain* OR restrict* OR implement* OR uptake OR adopt* OR adapt* OR accept* OR react* OR appropr* OR feasib* OR fidelity OR sustain* OR modification OR scale-up OR scaling-up OR scale up OR scale-out OR expan* OR replica* OR exten* OR institutionali?ation OR maintain OR continue*) AND TS= (africa OR nigeria OR ethiopia OR egypt OR  "DR congo" OR tanzania OR "south africa" OR kenya OR uganda OR algeria OR sudan OR morocco OR angola OR mozambique OR ghana OR madagascar OR cameroon OR "côte d'Ivoire" OR niger OR "burkina faso" OR mali OR malawi OR zambia OR Senegal OR chad OR Somalia OR zimbabwe OR guinea OR rwanda OR benin OR burundi OR tunisia OR "south sudan" OR  togo OR "sierra leone" OR libya OR congo OR liberia OR "central african republic" OR mauritania OR eritrea OR namibia OR gambia OR  botswana OR gabon OR lesotho OR "guinea-bissau" OR "equatorial guinea" OR mauritius OR eswatini OR djibouti OR comoros OR "cabo verde" OR "sao tome" OR seychelles) NOT TS= (pmtct OR mtct OR mother OR pregnant OR pregnancy OR lactating OR children OR adolescents OR "people who inject drugs" OR MSM OR "transgender persons" OR "sex workers" OR prisoners OR migrants) | 331 |
| **Global Health** | ((( (africa OR nigeria OR ethiopia OR egypt OR "DR congo" OR tanzania OR "south africa" OR kenya OR uganda OR algeria OR sudan OR morocco OR angola OR mozambique OR ghana OR madagascar OR cameroon OR "côte d'Ivoire" OR niger OR "burkina faso" OR mali OR malawi OR zambia OR Senegal OR chad OR Somalia OR zimbabwe OR guinea OR rwanda OR benin OR burundi OR tunisia OR "south sudan" OR togo OR "sierra leone" OR libya OR congo OR liberia OR "central african republic" OR mauritania OR eritrea OR namibia OR gambia OR botswana OR gabon OR lesotho OR "guinea-bissau" OR "equatorial guinea" OR mauritius OR eswatini OR djibouti OR comoros OR "cabo verde" OR "sao tome" OR seychelles))) AND (((experience* OR attitude* OR perception* OR learning OR Barrie* OR challeng* OR facilitator* OR enabler* OR benefit* OR success* OR constrain* OR difficult* OR enhanc* OR influen* OR interfer* OR motivat* OR obstruct* OR problem* OR promot* OR restrain* OR restrict* OR implement* OR uptake OR adopt* OR adapt* OR accept* OR react* OR appropr* OR feasib* OR fidelity OR sustain* OR modification OR scale-up OR scaling-up OR scale up OR scale-out OR expan* OR replica* OR exten* OR institutionali?ation OR maintain OR continue*))) AND ((("patient-centred care" OR "patient-centered care" OR "community supported models" OR "adherence club*" OR "task shifting" OR "community ART distribution" OR "community ART delivery" OR "community ART refill" OR "community client lead ART-delivery" OR "facility fast track" OR "quick pick-up" OR "differentiated care" OR "differentiated service" OR "differentiated intervention" OR "decentrali?ed care" OR "decentrali?ed service" OR "decentrali?ed intervention" OR "community care" OR "community service" OR "community intervention" OR "differentiated model*" OR down-referr* OR out-of-clinic))) AND ((("human immunodeficiency virus" OR "human immunodeficiency virus infection" OR HIV OR "antiretroviral treatment" OR "antiretroviral therapy" OR "antiretroviral therapy, highly active" OR "highly active antiretroviral therapy" OR HAART OR ART) )) AND (((patient* OR client* OR provider*) ))) NOT (((pmtct OR mtct OR mother OR pregnant OR pregnancy OR lactating OR children OR adolescents OR "people who inject drugs" OR MSM OR "transgender persons" OR "sex workers" OR prisoners OR migrants) )) | 108 |
| **CINAHL** | (patient* OR client* OR provider*) AND ("human immunodeficiency virus" OR "human immunodeficiency virus infection" OR HIV OR "antiretroviral treatment" OR "antiretroviral therapy" OR "antiretroviral therapy, highly active" OR "highly active antiretroviral therapy" OR HAART OR ART) AND ("patient-centred care" OR "patient-centered care" OR "community supported models" OR "adherence club*" OR "task shifting" OR "community ART distribution" OR "community ART delivery" OR "community ART refill" OR "community client lead ART-delivery" OR "facility fast track" OR "quick pick-up" OR "differentiated care" OR "differentiated service" OR "differentiated intervention" OR "decentrali?ed care" OR "decentrali?ed service" OR "decentrali?ed intervention" OR "community care" OR "community service" OR "community intervention" OR "differentiated model*" OR down-referr* OR out-of-clinic) AND (experience* OR attitude* OR perception* OR learning OR Barrie* OR challeng* OR facilitator* OR enabler* OR benefit* OR success* OR constrain* OR difficult* OR enhanc* OR influen* OR interfer* OR motivat* OR obstruct* OR problem* OR promot* OR restrain* OR restrict* OR implement* OR uptake OR adopt* OR adapt* OR accept* OR react* OR appropr* OR feasib* OR fidelity OR sustain* OR modification OR scale-up OR scaling-up OR scale up OR scale-out OR expan* OR replica* OR exten* OR institutionali?ation OR maintain OR continue*) AND (africa OR nigeria OR ethiopia OR egypt OR  "DR congo" OR tanzania OR "south africa" OR kenya OR uganda OR algeria OR sudan OR morocco OR angola OR mozambique OR ghana OR madagascar OR cameroon OR "côte d'Ivoire" OR niger OR "burkina faso" OR mali OR malawi OR zambia OR Senegal OR chad OR Somalia OR zimbabwe OR guinea OR rwanda OR benin OR burundi OR tunisia OR "south sudan" OR  togo OR "sierra leone" OR libya OR congo OR liberia OR "central african republic" OR mauritania OR eritrea OR namibia OR gambia OR  botswana OR gabon OR lesotho OR "guinea-bissau" OR "equatorial guinea" OR mauritius OR eswatini OR djibouti OR comoros OR "cabo verde" OR "sao tome" OR seychelles) NOT (pmtct OR mtct OR mother OR pregnant OR pregnancy OR lactating OR children OR adolescents OR "people who inject drugs" OR MSM OR "transgender persons" OR "sex workers" OR prisoners OR migrants) | 132 |
